# The relationship between obstructive sleep apnea and circulating tau levels: A meta‐analysis

**DOI:** 10.1002/brb3.2972

**Published:** 2023-03-20

**Authors:** Zhi‐Wei Huang, Hui‐Xue Zeng, Ya‐Ping Huang, Tie‐Zhu Wang, Wen‐Sen Huang, Yan‐Fei Huang, Li Lin, Hao Li

**Affiliations:** ^1^ Department of Otolaryngology Quanzhou First Hospital Affiliated to Fujian Medical University Quanzhou Fujian Province People's Republic of China; ^2^ Department of Respiratory and Critical Care Medicine Zhangzhou Affiliated Hospital of Fujian Medical University Zhangzhou Fujian Province People's Republic of China

**Keywords:** Alzheimer's disease, meta‐analysis, obstructive sleep apnea, tau

## Abstract

**Background:**

Alzheimer's disease (AD) is an irreversible, progressive brain disorder that impairs memory, thinking, language, and, eventually, the ability to carry out the simplest of tasks. Tau protein, the major component of neurofibrillary tangles, is considered a key mediator of AD pathogenesis. The association between obstructive sleep apnea (OSA) and circulating tau remains unclear. The aim of the present meta‐analysis was to evaluate the relationship between OSA and circulating tau via quantitative analysis.

**Methods:**

A systematic search of Pubmed, Embase, and Web of Science were performed. The mean values of circulating total tau (T‐tau) and phosphorylated tau (P‐tau) in OSA and control groups were extracted. Standardized mean difference (SMD) with 95% confidence interval (CI) was calculated by using a random‐effect model or fixed‐effect model.

**Results:**

A total of seven studies comprising 233 controls and 306 OSA patients were included in this study. The meta‐analysis showed that the circulating T‐tau level was significantly higher in OSA patients than those in the control group (SMD = 1.319, 95% CI = 0.594 to 2.044, *z* = 3.56, *p* < .001). OSA patients also had significantly higher circulating P‐tau level than control group (SMD = 0.343, 95% CI = 0.122 to 0.564, *z* = 3.04, *p* = .002).

**Conclusions:**

The present meta‐analysis demonstrated that both circulating T‐tau and P‐tau levels were significantly increased in OSA subjects when compared with non‐OSA subjects. Larger sample‐size studies on the association between OSA and circulating tau are still required to further validate our results.

## Graphical abstract

The present meta‐analysis demonstrated that circulating tau level was significantly increased in OSA subjects when compared with non‐OSA subjects.

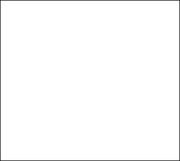

## 1 INTRODUCTION

Obstructive sleep apnea (OSA) is a highly prevalent breathing‐related sleep disorder, characterized by intermittent and repeated episodes of collapse of the upper airway during sleep. These episodes are associated with intermittent hypoxia and sleep fragmentation, both of which can raise systematic inflammation, oxidative stress, and sympathetic activity. The prevalence of OSA increases with age and ranges 30%–80% in elderly subjects (Heinzer et al., 2016; Zamarron et al., 1999). Old age is the most important risk factor for Alzheimer's disease (AD). Increasing evidence also supports a link between OSA and increased risk of cognitive decline, particularly AD (Bubu et al., 2020).

AD is an irreversible, progressive brain disorder that impairs memory, thinking, language, and, eventually, the ability to carry out the simplest of tasks. AD is the most common cause of dementia among neurodegenerative diseases and affects approximately 10% of individuals older than 65 years (Blennow et al., 2006). Neurofibrillary tangles (NFTs) and neuritic plaques are the classical neuropathological hallmarks of AD. Tau protein, the major component of NFTs, is considered a key mediator of AD pathogenesis. Tau protein aggregation is a long‐term process, which starts 20 years before any noticeable symptoms (Kametani & Hasegawa, 2018). Robust evidence has suggested that circulating tau levels are correlated with the severity of AD (Ding et al., 2021; Mielke et al., 2017; Pase et al., 2019). This revealed that circulating tau is a promising biomarker for early diagnosis and prognostic prediction of AD.

The association between OSA and circulating tau remains unclear. The present study was conducted to quantitatively evaluate if there is a relationship between OSA and circulating tau.

## 2 METHODS

This meta‐analysis was conducted based on preferred reporting items for systematic reviews and meta‐analysis (PRISMA) guidelines (Moher et al., 2009).

### 2.1 Search strategy

A systematic search of the literature was conducted on PubMed, Embase, and Web of Science using the keywords “OSA” OR “sleep apnea” OR “sleep apnoea” OR “sleep‐disordered breathing” AND “tau” AND “blood” OR “serum” OR “plasma” OR “circulating” between 1990 and January 2023 without language restriction. A combination of free text terms and Mesh‐terms were used in the search. The reference lists of individual articles were searched to find other possibly relevant studies.

### 2.2 Inclusion/exclusion criteria of literature

The following inclusion criteria were applied: (1) All participants included in the study were adults with age ≥ 18 years; (2) the diagnosis of OSA was according to polysomnography with apnea‐hypopnea index (AHI) ≥ 5; (3) OSA subjects were newly diagnosed and did not receive any form of treatment; (4) circulating tau levels were reported both in OSA and control group. Exclusion criteria were: case reports, reviews, letters, editorials, conference articles, animal studies, duplicate publications, and those with insufficient data. If the essential data were not reported in the study, the authors were contacted via email. If there was no response following two communication attempts, the article was ruled out. When a disagreement about the inclusion of a study occurred, a third reviewer engaged.

### 2.3 Data extraction and quality assessment

The following data were extracted using a standardized form by two authors independently: first author, publication year, region of study, study design, sample size, OSA diagnosis method, time of blood sampling, population characteristics, sample type, and the mean value of total tau (T‐tau) and phosphorylated tau (P‐tau). The study quality was evaluated using the Newcastle–Ottawa Scale (NOS; Wells et al., 2009). The scale assesses methodology in three areas: selection, comparability, and exposure. The total score is 9, including 4 for the selection part, 2 for the comparability part, and 3 for the exposure part. A total score ≥ 7 indicates high quality.

### 2.4 Statistical analyses

The statistical analyses in this study were performed with STATA 12.0. Standardized mean difference (SMD) and 95% confidence interval (CI) were used to evaluate the differences in circulating tau levels between OSA and control groups. The heterogeneity of the included studies was assessed by the chi‐squared test and I^2^ statistic. When *p*  >  .05 or I^2^ <  50%, a fixed‐effect model was used for meta‐analysis; otherwise, a random‐effect model was used. To explore the possible sources of heterogeneity, sensitive analysis and subgroup analysis were conducted. Begg's and Egger's tests were used to check for publication bias. A *p* < .05 was considered statistically significant.

## 3 RESULTS

### 3.1 Searching results

**FIGURE 1:**
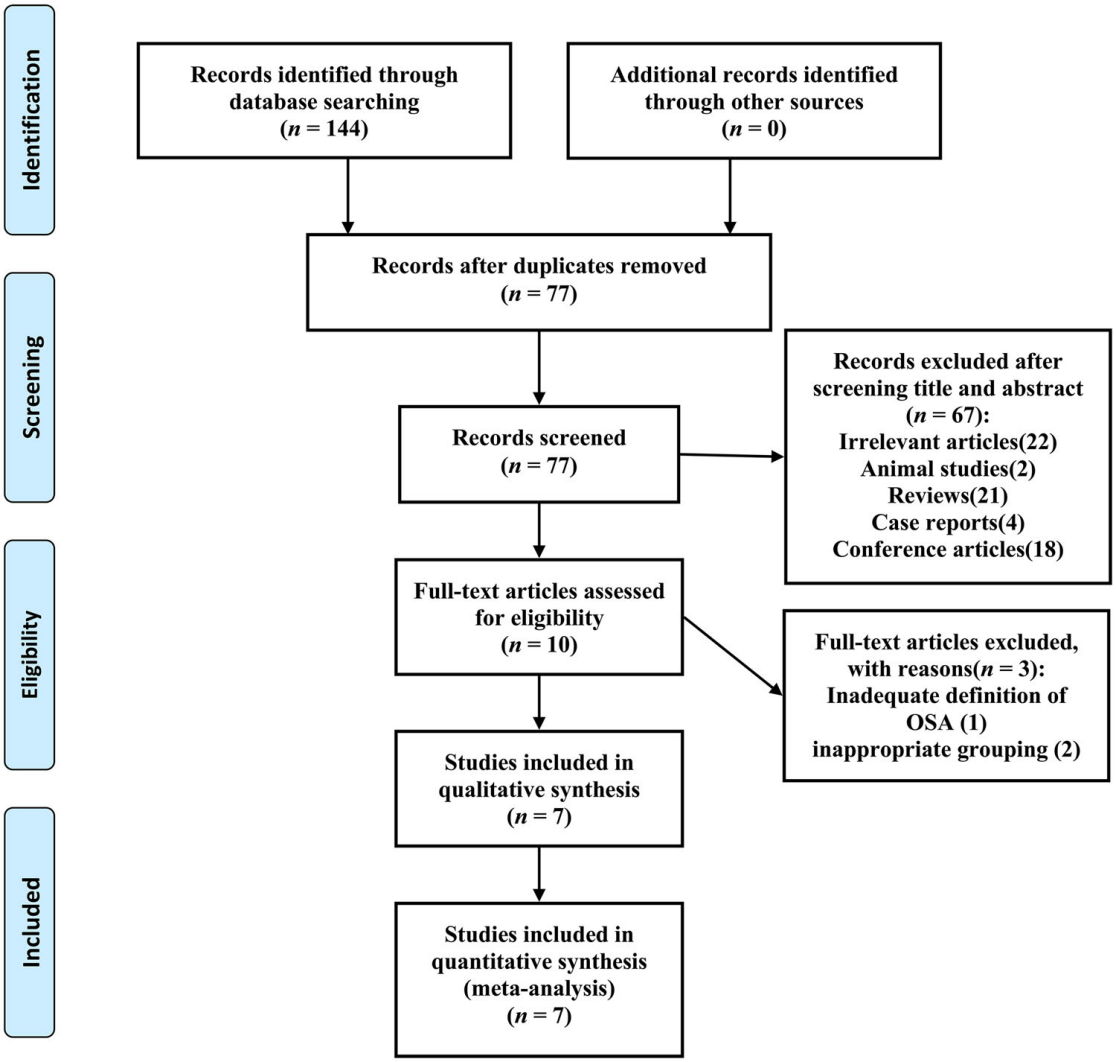
Flow diagram of study selection. OSA, obstructive sleep apnea.

A PRISMA flow diagram of the study selection process is presented in Figure 1. A total of 144 papers were identified from the initial electronic and manual search. Duplicates were excluded resulting in 77 papers. Sixty‐seven papers were further excluded after screening titles and abstracts, leaving 10 citations for full‐text review. Three papers were subsequently excluded for the following reasons: One study defined OSA using a questionnaire (Huang et al., 2021); another study divided patients into normal‐to‐moderate and severe OSA groups (Tsai, Liu, et al., 2022); one study did not group by OSA severity (Tsai, Wu, et al., 2022). At last, seven papers were considered eligible for the meta‐analysis.

### 3.2 Characteristics of the studies

A total of seven studies comprising 233 controls and 306 OSA patients were included in the meta‐analysis (Bhuniya et al., 2022; Bu et al., 2015; Chen et al., 2022; Kong et al., 2021; Motamedi et al., 2018; Pai et al., 2022; Sun et al., 2022). Among them, four studies with 138 controls and 206 OSA subjects compared P‐tau. All studies were cross‐sectional design. All the included subjects were free of neurological disorders. The majority of the subjects were male (73.1%). The mean age of the subjects ranged from 29.7 to 63.1 years old, body mass index (BMI) ranged from 23.02 to 33.2, and AHI in the OSA patient group ranged from 18.4 to 63.92. T‐tau was detected in plasma in five studies (Chen et al., 2022; Kong et al., 2021; Motamedi et al., 2018; Pai et al., 2022; Sun et al., 2022) and serum in two studies (Bhuniya et al., 2022; Bu et al., 2015). The NOS scores ranged from 6 to 9, indicating that the methodological quality was generally good (Table S1). The detailed characteristics of the included studies are presented in Table 1.

**TABLE 1:** Characteristics of the included studies.

**Study**		**Bu (2015)**	**Motamedi (2018)**	**Kong (2021)**	**Bhuniya (2022)**	**Pai (2022)**	**Sun (2022)**	**Chen (2022)**
Country		China	USA	China	India	China, Taiwan	China	China, Taiwan
Study design		Cross‐sectional study	Cross‐sectional study	Cross‐sectional study	Cross‐sectional study	Cross‐sectional study	Cross‐sectional study	Cross‐sectional study
NOS score		9	8	8	6	7	9	8
Clinical condition of patients		No family history of dementia; no neurologic disorder	No prior diagnosis of traumatic brain injury	No family history of dementia; no neurological diseases	Exclusion of neurological and psychiatric disorders	Normal cognition	No dementia	No major neurological or psychiatric disorders, head trauma
OSA diagnosis	Method	PSG	PSG	PSG	PSG	PSG	PSG	PSG
	events/h	AHI≥5	AHI ≥ 5	AHI≥5	AHI ≥ 5	AHI ≥ 5	AHI ≥ 5	OSA symptoms plus AHI ≥ 5 or solely AHI ≥ 15
Sample size/male (％)	OSA	45 (73.33)	50 (98)	3 5(85.8)	46 (95.7)	20 (0)	79 (69.6)	30 (90.0)
	NC	49 (69.39)	24 (92)	16 (87.5)	30 (83.3)	37 (37.8)	43 (69.8)	34 (50.0)
	* p* ‐value	.82	NR	.84	.1587	< .05	NR	.001
Age	OSA	44.31 ± 9.96	34.9 ± 7.9	39.18 ± 9.22	44.1 ± 12.4	40.3 ± 8.7	54.42 ± 6.98	41.93 ± 1.65
	NC	42.98 ± 9.62	30.9 ± 7.8	42.43 ± 9.79	29.7 ± 5.9	63.1 ± 12.4	54.61 ± 7.28	43.21 ± 2.25
	*p*‐value	.511	NR	.26	< .001	< .001	NR	.65
BMI (kg/m^2^)	OSA	26.84 ± 2.52	30.8 ± 4.2	25.65 ± 2.78	33.2 ± 8.9	NR	28.01 ± 4.52	26.18 ± 0.52
	NC	26.07 ± 2.61	28.6 ± 3.8	23.02 ± 2.70	24 ± 1.5	NR	26.23 ± 4.16	24.83 ± 0.54
	*p* ‐value	.146	NR	.003	< .001	NR	NR	.08
AHI (events/h)	OSA	32.68 ± 23.34	18.4 ± 18.2	63.92 ± 23.65	NR	54.6 ± 17.5	36.87 ± 20.89	41.93 ± 4.33
	NC	2.26 ± 1.35	2.1 ± 1.3	2.62 ± 1.93	NR	NR	2.81 ± 1.18	2.68 ± 0.29
	*p*‐value	< .001	< .001	< .001	NR	NR	NR	< .001
Sample type		Serum	Plasma	Plasma	Serum	Plasma	Plasma exosomes	Plasma
Time of blood sampling		Fasting blood; 06:00–07:00 a.m.	9:00 a.m.–12:00 p.m.	Fasting blood	Fasting blood; 08:00−09:00 a.m.	NR	Fasting blood; 06:00–07:00 a.m.	10:00−11:30 a.m.
Assay		ELISA	ELISA	ELISA	ELISA	Immunomagnetic reduction assays	ELISA	Immunomagnetic reduction technology
T‐tau	OSA	45.4 ± 34.24 pg/mL	4.32 ± 2.62 pg/mL	11.88 ± 7.05 pg/mL	177.14 ± 64.74 pg/mL	20.17 ± 2.77 pg/mL	160.52 ± 34.01 pg/mL	21.43 ± 0.55 pg/mL
	NC	40.49 ± 24.61 pg/mL	2.48 ± 1.94 pg/mL	7.64 ± 4.17 pg/mL	70.99 ± 68.86 pg/mL	15.13 ± 3.62 pg/mL	142.47 ± 12.53 pg/mL	18.27 ± 0.85 pg/mL
P‐tau	OSA	42.03 ± 21.58 pg/mL	NR	26.31 ± 14.41 pg/mL	11.95 ± 12.37 pg/mL	NR	44.15 ± 13.02 pg/mL	NR
	NC	30.56 ± 17.3 pg/mL	NR	17.34 ± 9.12 pg/mL	12.91 ± 10.98 pg/mL	NR	40.53 ± 11.28 pg/mL	NR

Abbreviations: AHI, apnea‐hypopnea index; ELISA, enzyme‐linked immunosorbent assay; NC, normal control; NR, not reported; NS, not significant; OSA, obstructive sleep apnea; PSG, polysomnography; P‐tau, phosphorylated tau; T‐tau, total tau.

### 3.3 Pooled analysis of T‐tau

**FIGURE 2:**
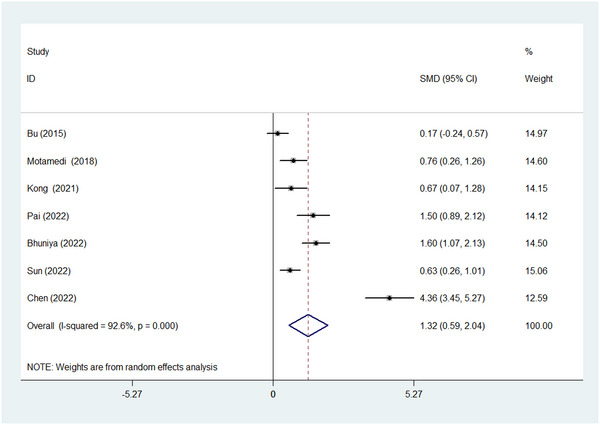
Forest plot of comparison of total tau (T‐tau) between obstructive sleep apnea (OSA) group and control group. CI, confidence interval; SMD, standardized mean difference.

As a high heterogeneity existed between studies (chi‐squared = 81.10, *p* < .001; I^2^ = 92.6%); thus, a random‐effect model was conducted to pool analysis. As shown in Figure 2, circulating T‐tau level was significantly higher in OSA patients than those in the control group (SMD = 1.319, 95% CI = 0.594 to 2.044, *z* = 3.56, *p* < .001).

### 3.4 Pooled analysis of P‐tau

**FIGURE 3:**
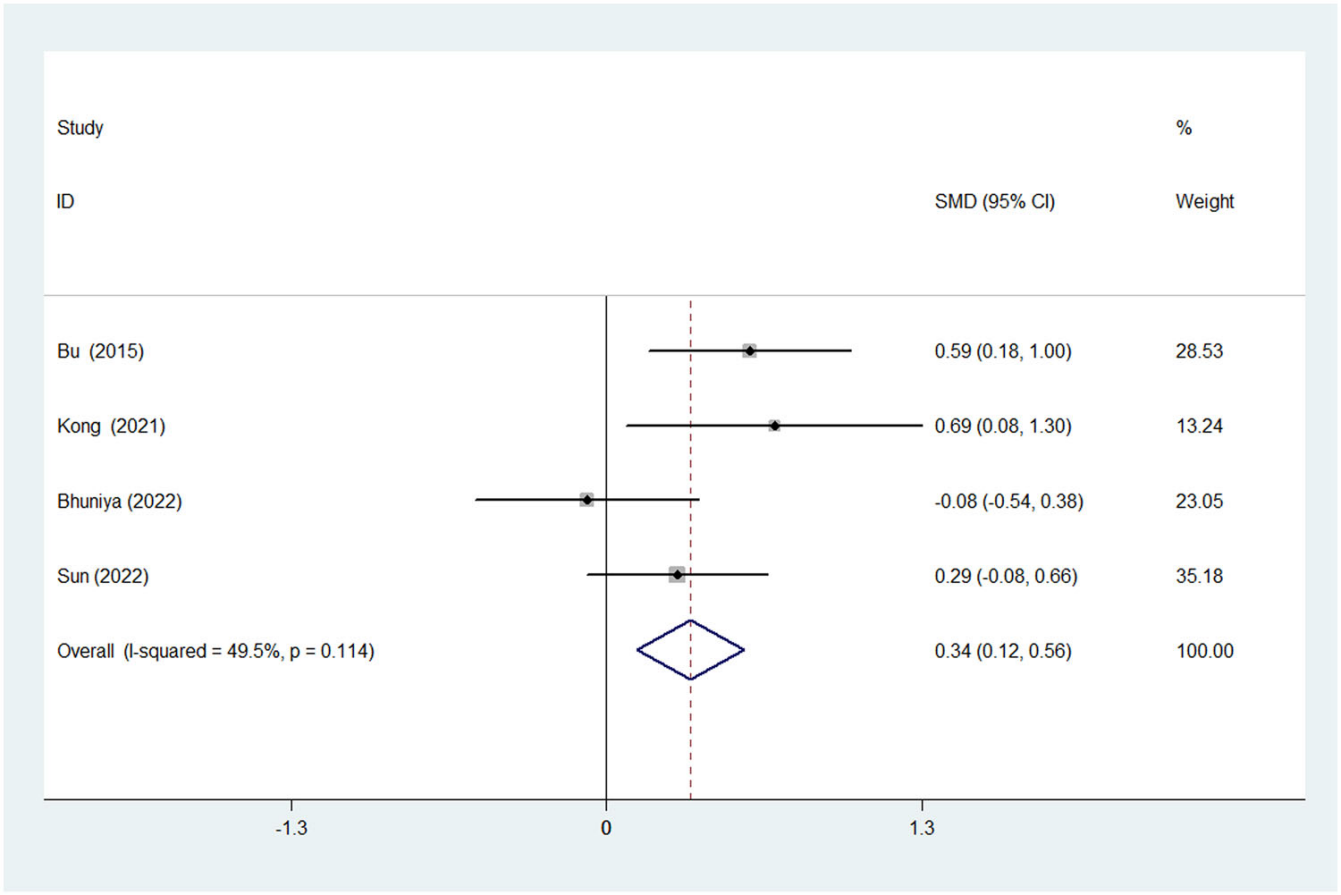
Forest plot of comparison of phosphorylated tau (P‐tau) between OSA group and control group. CI, confidence interval; SMD, standardized mean difference.

No significant heterogeneity (chi‐squared = 5.95, *p* = .114; I^2^ = 49.5%) was observed among studies and a fixed‐effect model was applied. The pooled analysis revealed that OSA patients had significantly higher circulating P‐tau level than the control group (SMD = 0.343, 95% CI = 0.122 to 0.564, *z* = 3.04, *p* = .002; Figure 3).

### 3.5 Sensitivity analysis and subgroup analyses

**FIGURE 4:**
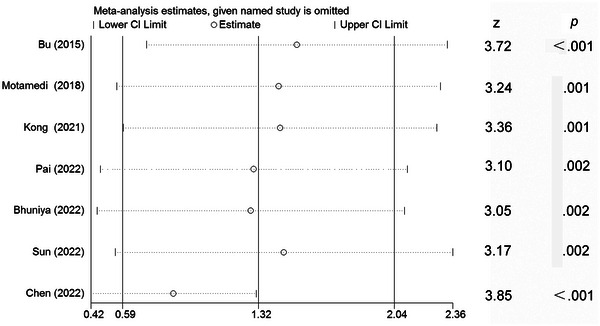
The sensitivity analysis by omitting one study at each turn. CI, confidence interval.

As I^2^ was high for pooling the data of T‐tau, sensitivity analysis was performed. Sensitivity analysis showed that the results were not materially altered after sequentially excluding each study, confirming the robustness of the results (Figure 4). Subgroup analyses based on age (< 50 and ≥ 50), BMI (< 27 and ≥ 27), severity of OSA (AHI < 50 and ≥ 50), NOS scores (< 8 and ≥ 8), and sample size (< 70 and ≥ 70) were further conducted. The subgroup analyses showed that the differences in age, BMI, the severity of OSA, NOS scores, and sample size did not affect the relationship between OSA and circulating T‐tau (Table 2).

**TABLE 2:** The results of subgroup analyses for T‐tau

**Subgroup**	**Number of studies**	**Heterogeneity**			**SMD**			
		**X^2^**	***p***	**I^2^(％)**	**SMD**	**95％CI**	***z***	***p***
NOS scores								
< 8	2	0.05	.818	0	1.559	1.159–1.958	2.57	.010
≥ 8	5	68.67	< .001	94.2	1.240	0.296–2.185	7.65	< .001
Age								
< 50	5	75.33	< .001	94.7	1.455	0.386–2.525	2.67	.008
≥ 50	2	5.60	.018	82.2	1.035	0.185–1.884	2.39	.017
BMI								
< 27	4	68.66	< .001	95.6	1.388	0.151–2.624	2.20	.028
≥ 27	2	5.11	.024	80.4	1.175	0.352–1.998	2.80	.005
AHI								
< 50	4	68.61	< .001	95.6	1.400	0.222–2.578	2.33	.020
≥ 50	2	3.58	.058	72.1	1.087	0.271–1.902	2.61	.009
Sample size								
< 70	4	18.00	< .001	83.3	0.771	0.222–1.319	2.76	.006
≥ 70	3	44.14	< .001	95.5	2.147	0.265–4.028	2.24	.025

Abbreviations: AHI, apnea‐hypopnea index; BMI, body mass index; CI, confidence interval; NOS, Newcastle–Ottawa Scale; SMD, standardized mean difference; T‐tau, total tau.

### 3.6 Publication bias

**FIGURE 5:**
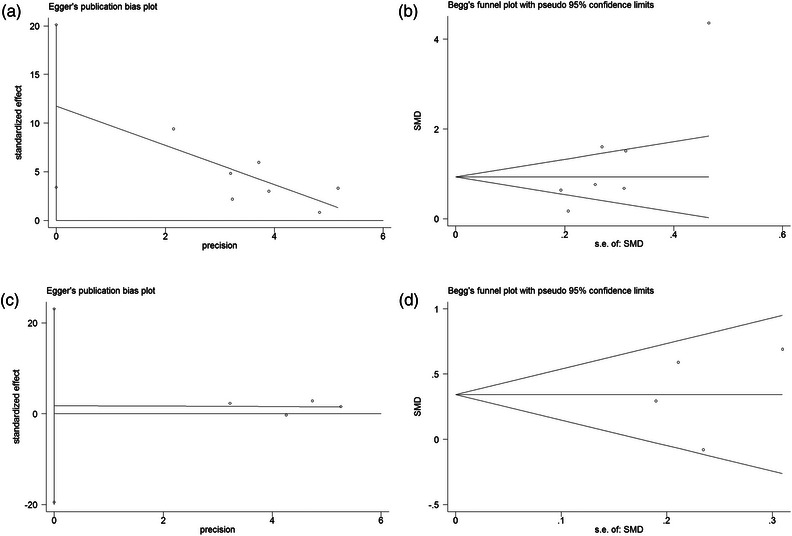
The funnel plot for exploring the publication bias by using Egger's tests (A. T‐tau; C. P‐tau) and Begg's tests (B. T‐tau; D. P‐tau). CI, confidence interval; SMD, standardized mean difference.

For circulating T‐tau, Egger's tests (*p* = .015) suggested evidence of publication bias, while Begg's tests (*p* = .072) showed no evidence to support publication bias (Figure 5A,B). For circulating P‐tau, both Egger's tests (*p* = .754) and Begg's (*p* = 1.000) proved no evidence of publication bias in our study (Figure 5C,D).

## 4 DISCUSSION

The results of this meta‐analysis demonstrated that OSA was significantly and positively correlated with circulating T‐tau and P‐tau in a cognitively normal population. Both circulating T‐tau and P‐tau of the OSA group were significantly higher than that of the control group.

There is increasing literature to suggest that OSA is not only highly prevalent among AD patients but also increases the risk of developing AD and even dementia (Bubu et al., 2020; Lee et al., 2019; Yaffe et al., 2011). Lee et al. (2019) studied nationwide health check‐up cohort data between 2002 and 2015 and found that OSA patients were almost 1.58 times more likely to develop AD than those without OSA after adjusting for possible confounding factors. A prospective study followed up 298 older adult women without dementia at baseline for 5 years. Multivariate logistic regression showed that OSA was significantly associated with an increased risk of developing mild cognitive impairment (MCI) or dementia after adjusting for confounding variables (Yaffe et al., 2011). Another study reported that OSA was associated with an earlier age at the onset of MCI or AD‐dementia and continuous positive airway pressure (CPAP) treatment delayed the age at MCI onset (Osorio et al., 2015). A prospective matched‐control cohort study utilized data from Taiwan's Health Insurance Database comprising 1414 patients and demonstrated gender‐dependent, age‐dependent, and time‐dependent associations between OSA and dementia (Chang et al., 2013).

Some studies evaluated the effect of OSA treatment on AD/dementia (Ancoli‐Israel et al., 2008; Cooke, Ancoli‐Israel, et al., 2009; Cooke, Ayalon, et al., 2009). Cooke, Ancoli‐Israel et al. (2009) performed a randomized placebo‐controlled trial and found that CPAP therapy resulted in deeper sleep after just one night, with improvements maintained for 3 weeks. Then they further studied a subset of patients with long‐term CPAP use and patients who discontinued CPAP treatment and reported that 13 months of CPAP treatment might result in slowing of cognitive deterioration for patients with AD and OSA (Cooke, Ayalon, et al., 2009). Another randomized controlled study proved significant cognitive improvements in AD patients induced by CPAP treatment. The improvements were mainly in episodic verbal learning, memory, and executive functioning (Ancoli‐Israel et al., 2008). The above studies demonstrated a positive effect of CPAP treatment on OSA patients with AD. This further supported that OSA was an important risk factor for AD and dementia.

The role of circulating tau proteins as dementia biomarkers has been validated by accumulating evidence (Chiu et al., 2014; Mattsson et al., 2016; Mielke et al., 2017). In 2014, Chiu et al. (2014) reported that MCI or early AD patients had significantly elevated plasma tau levels when compared with healthy controls. A prospective study including 1284 subjects suggested that higher plasma tau was associated with AD dementia. Circulating tau levels were also correlated with longitudinal changes in neuroimaging parameters and cognition. Mielke et al. (2017) followed up 458 participants for at least 1 year. The results showed that the higher levels of plasma T‐tau were correlated with significant declines in attention, memory, global cognition, and visuospatial ability over 3 years of follow‐up.

Our study suggested that OSA was significantly and positively correlated with circulating T‐tau and P‐tau levels in a group of cognitively normal patients. The finding has some clinical implications. First, OSA confers an increased risk of developing AD and dementia. Intermittent hypoxia, sleep fragmentation, reduced slow‐wave sleep, and intrathoracic pressure swings are suggested to be possible mechanisms by which OSA induces neurodegenerative changes (Bubu et al., 2020). However, the detailed pathophysiology of this relationship has not been well defined. Establishing a link between OSA and biomarkers of dementia is helpful in advancing our understanding of the pathophysiological mechanisms involved in neurodegeneration. Second, as tau protein aggregation starts 20 years before any noticeable symptoms, tau is served as a promising biomarker for early diagnosis and prognostic prediction. The finding of our study implies that OSA may predispose patients to early cognitive impairments. Therefore, it is suggested that OSA patients should be closely monitored for early signs of cognitive impairment, and early treatment should be instituted to prevent the progression of neurodegeneration. Our finding also raised the question of whether treatment of OSA could delay the age at MCI onset in OSA patients by improving tau metabolism. Future studies are needed to clarify it.

Several limitations must be addressed when interpreting these results. First, although a significant relationship between OSA and elevated circulating T‐tau and P‐tau levels was identified, the strength of evidence might be weak because of the limited sample size. Second, subgroup analysis based on some confounding variables cannot be performed because of the limited number of included studies. Third, as the severity of OSA was not grouped in most studies, the dose–response relationship between OSA and elevated circulating tau levels cannot be evaluated in this study. Fourth, the circulating T‐tau level varies significantly in different studies. The variability could be explained by several factors: differences of blood sampling time, sample type, measure assay, and patient characteristics. The SMD was applied to estimate the pooled results. Finally, all studies were cross‐sectional in design, so we cannot explore a causal relationship.

In conclusion, the present meta‐analysis demonstrated that both circulating T‐tau and P‐tau levels were significantly increased in OSA subjects when compared with non‐OSA subjects. The results of our study support the potential role of circulating tau in linking OSA with increased risk of AD and even dementia. However, larger sample‐size studies on the association between OSA and circulating tau are still required to further validate our results.

## CONFLICT OF INTEREST STATEMENT

The authors have disclosed no conflicts of interest.

### PEER REVIEW

The peer review history for this article is available at https://publons.com/publon/10.1002/brb3.2972.

## Supporting information

TABLE S1 Quality assessment of case‐control studies with the Newcastle–Ottawa Scale.

## Data Availability

The data that support the findings of this study are available from the corresponding author upon reasonable request.
