# Supplementary material for: The relationship between obstructive sleep apnea and circulating tau levels: A meta‐analysis
Source: Brain Behav. 2023 Mar 20;13(4):e2972. doi: 10.1002/brb3.2972 (PMC10097049; doi:10.1002/brb3.2972)
Supplement: Supplementary file 1 — TABLE S1 Quality assessment of case‐control studies with the Newcastle–Ottawa Scale. [file BRB3-13-e2972-s001.docx]

| Studies | Selection | | | | Comparability | Exposure | | | Total score |
| --- | --- | --- | --- | --- | --- | --- | --- | --- | --- |
|  | Adequate case definition | Representativeness of the cases | Selection of Controls | Definition of Controls | Comparability of cases and controls | Ascertainment of exposure | Same method of ascertainment for cases and controls | Non-Response rate |  |
| Bu 2015 | 1 | 1 | 1 | 1 | 2 | 1 | 1 | 1 | 9 |
| Motamedi 2018 | 1 | 0 | 1 | 1 | 2 | 1 | 1 | 1 | 8 |
| Kong 2021 | 1 | 1 | 1 | 1 | 1 | 1 | 1 | 1 | 8 |
| Bhuniya 2022 | 1 | 1 | 0 | 1 | 0 | 1 | 1 | 1 | 6 |
| Pai 2022 | 1 | 1 | 1 | 1 | 0 | 1 | 1 | 1 | 7 |
| Sun 2022 | 1 | 1 | 1 | 1 | 2 | 1 | 1 | 1 | 9 |
| Chen 2022 | 1 | 1 | 1 | 1 | 1 | 1 | 1 | 1 | 8 |

Table S1: Quality assessment of case-control studies with the Newcastle–Ottawa Scale
